# 1-Cyano­meth­yl-1,4-diazo­niabicyclo­[2.2.2]octane tetra­bromidocuprate(II)

**DOI:** 10.1107/S1600536810023469

**Published:** 2010-06-23

**Authors:** Ying Cai

**Affiliations:** a Ordered Matter Science Research Center, Southeast University, Nanjing 211189, People’s Republic of China

## Abstract

In the crystal structure of the title complex, (C_8_H_15_N_3_)[CuBr_4_], the Cu atom is coordinated by four bromido ligands within a strongly distorted tetra­hedron. The anions and cations are connected by weak N—H⋯Br and C—H⋯Br hydrogen-bonding inter­actions.

## Related literature

For the uses of DABCO (1,4-diaza­bicyclo­[2.2.2]octa­ne) and its derivatives, see: Basaviah *et al.* (2003 ▶); Chen *et al.* (2010 ▶).
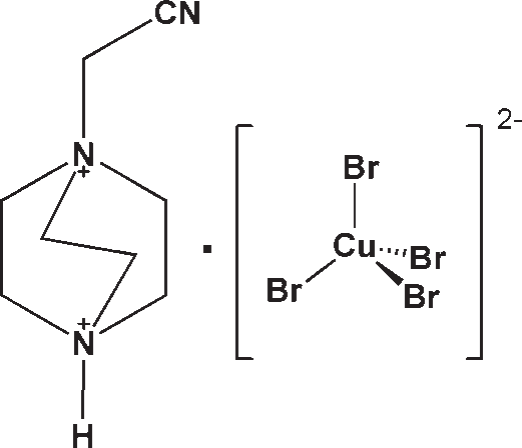


## Experimental

### 

#### Crystal data

(C_8_H_15_N_3_)[CuBr_4_]
*M* *_r_* = 536.41
Monoclinic, 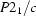

*a* = 8.4793 (17) Å
*b* = 13.911 (3) Å
*c* = 12.506 (3) Å
β = 97.75 (3)°
*V* = 1461.7 (5) Å^3^
*Z* = 4
Mo *K*α radiation
μ = 12.41 mm^−1^
*T* = 293 K
0.3 × 0.3 × 0.2 mm

#### Data collection

Rigaku Mercury CCD diffractometer
Absorption correction: multi-scan (*CrystalClear*; Rigaku, 2005 ▶) *T*_min_ = 0.041, *T*_max_ = 0.092
14798 measured reflections
3347 independent reflections
2642 reflections with *I* > 2σ(*I*)
*R*_int_ = 0.069

#### Refinement

*R*[*F* ^2^ > 2σ(*F* ^2^)] = 0.046
*wR*(*F* ^2^) = 0.099
*S* = 1.10
3347 reflections
145 parameters
H-atom parameters constrained
Δρ_max_ = 1.48 e Å^−3^
Δρ_min_ = −0.93 e Å^−3^

### 

Data collection: *CrystalClear* (Rigaku, 2005 ▶); cell refinement: *CrystalClear*; data reduction: *CrystalClear*; program(s) used to solve structure: *SHELXS97* (Sheldrick, 2008 ▶); program(s) used to refine structure: *SHELXL97* (Sheldrick, 2008 ▶); molecular graphics: *SHELXTL* (Sheldrick, 2008 ▶); software used to prepare material for publication: *SHELXL97*.

## Supplementary Material

Crystal structure: contains datablocks I, global. DOI: 10.1107/S1600536810023469/nc2190sup1.cif

Structure factors: contains datablocks I. DOI: 10.1107/S1600536810023469/nc2190Isup2.hkl

Additional supplementary materials:  crystallographic information; 3D view; checkCIF report

## Figures and Tables

**Table 1:** Hydrogen-bond geometry (Å, °)

*D*—H⋯*A*	*D*—H	H⋯*A*	*D*⋯*A*	*D*—H⋯*A*
N3—H3*C*⋯Br3^i^	0.96	2.62	3.420 (5)	142
N3—H3*C*⋯Br2^i^	0.96	2.95	3.545 (5)	122
C4—H4*A*⋯Br3^i^	0.97	2.92	3.555 (6)	124
N3—H3*C*⋯Br4	0.96	2.86	3.406 (5)	117
C2—H2*A*⋯Br1^ii^	0.97	2.91	3.638 (6)	132
C2—H2*B*⋯Br4^iii^	0.97	2.73	3.608 (6)	150

Symmetry codes: (i) 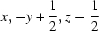; (ii) 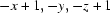; (iii) 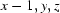.

**Fig. 1.**
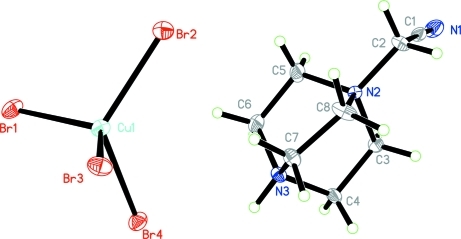
Crystal structure of the title compound with labelling and displacement ellipsoids drawn at the 30% probability level.

**Fig. 2.**
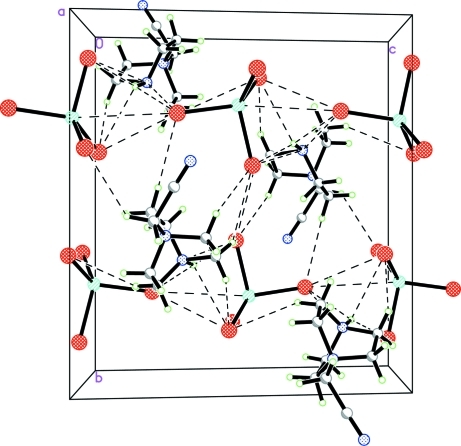
Crystal structure of the title compound with view along the a axis. Intermolecular interactions are shown as dashed lines.

## supplementary crystallographic information

### Comment

1,4-Diazabicyclo[2.2.2]octane (DABCO) is used as a good organocatalyst for a large number of reactions because of its nucleophilicity (Basaviah *et al.*, 2003) and some of its derivatives are ferroelectrics (Chen *et al.*, 2010). The structure determination of the title compound was performed within a project on the electric properties of 1,4-Diazabicyclo[2.2.2]octane derivatives. Within this project the crystals were obtained by accident.

The asymmetric unit of the title compound, (I), is shown in Fig. 1. The Cu atoms are coordinated by four Br atoms with very similar distances in the range of 2.36 (1) to 2.41 (4) Å. The Br—Cu—Br bond angles are between 97.32 (4) and 126.31 (4)° which shows that the coordination polyhedron can be described as a strongly disotorted tetrahedron. The (C_8_H_14_N_3_)^2+^ cations are connected to the CuBr_4_^2-^ anions *via* very weak intermolecular interactions (Fig. 2 and Table 1).

### Experimental

1,4-Diaza-bicyclo[2.2.2]octane (dabco) (0.05 mol, 5.6 g) and bromoacetonitrile (0.1 mol, 12.00 g) were dissolved in CH_3_CN (40 ml) with stirring for 1 h at room temperature. 1-(cyanomethyl)-4-aza-1-azonia-bicyclo[2.2.2]octane bromide quickly formed as a white solid was filtered, washed with acetonitrile and dried (yield: 80%).

CuBr_2 _(0.001 mol, 0.223 g) and 4 ml 60% HBr were dissolved in MeOH (20 ml) and 1-(cyanomethyl)-4-aza-1-azonia-bicyclo[2.2.2]octane bromide (0.002 mol, 0.464 g) dissolved in 10 ml of methanol was added. The mixture was stirred until a clear solution was obtained. After slow evaporation of the solvent, colourless plate crystals of the title compand suitable for X-ray analysis were obtained in about 68% yield.

### Refinement

H atoms bound to carbon and nitrogen were placed in idealized positions [C—H = 0.97 Å and N—H = 0.96 Å] and allowed to ride on their parent atoms with *U*_iso_ fixed at 1.2 *U*_eq_(C,*N*).

### Crystal data

**Table:**

(C_8_H_15_N_3_)[CuBr_4_]	*F*(000) = 1012
*M**_r_* = 536.41	*D*_x_ = 2.438 Mg m^−^^3^
Monoclinic, *P*2_1_/*c*	Mo *K*α radiation, λ = 0.71073 Å
Hall symbol: -P 2ybc	Cell parameters from 3450 reflections
*a* = 8.4793 (17) Å	θ = 6.2–55.3°
*b* = 13.911 (3) Å	µ = 12.41 mm^−^^1^
*c* = 12.506 (3) Å	*T* = 293 K
β = 97.75 (3)°	Block, brown
*V* = 1461.7 (5) Å^3^	0.3 × 0.3 × 0.2 mm
*Z* = 4	

### Data collection

**Table:**

Rigaku Mercury CCD diffractometer	3347 independent reflections
Radiation source: fine-focus sealed tube	2642 reflections with *I* > 2σ(*I*)
graphite	*R*_int_ = 0.069
ω scans	θ_max_ = 27.5°, θ_min_ = 3.1°
Absorption correction: multi-scan (*CrystalClear*; Rigaku, 2005)	*h* = −11→10
*T*_min_ = 0.041, *T*_max_ = 0.092	*k* = −18→17
14798 measured reflections	*l* = −16→16

### Refinement

**Table:**

Refinement on *F*^2^	Secondary atom site location: difference Fourier map
Least-squares matrix: full	Hydrogen site location: inferred from neighbouring sites
*R*[*F*^2^ > 2σ(*F*^2^)] = 0.046	H-atom parameters constrained
*wR*(*F*^2^) = 0.099	*w* = 1/[σ^2^(*F*_o_^2^) + (0.0321*P*)^2^ + 5.2474*P*] where *P* = (*F*_o_^2^ + 2*F*_c_^2^)/3
*S* = 1.10	(Δ/σ)_max_ < 0.001
3347 reflections	Δρ_max_ = 1.48 e Å^−^^3^
145 parameters	Δρ_min_ = −0.93 e Å^−^^3^
0 restraints	Extinction correction: *SHELXS*
Primary atom site location: structure-invariant direct methods	Extinction coefficient: 0.0476 (15)

### Special details

**Table:**

Geometry. All e.s.d.'s (except the e.s.d. in the dihedral angle between two l.s. planes) are estimated using the full covariance matrix. The cell e.s.d.'s are taken into account individually in the estimation of e.s.d.'s in distances, angles and torsion angles; correlations between e.s.d.'s in cell parameters are only used when they are defined by crystal symmetry. An approximate (isotropic) treatment of cell e.s.d.'s is used for estimating e.s.d.'s involving l.s. planes.
Refinement. Refinement of *F*^2^ against ALL reflections. The weighted *R*-factor *wR* and goodness of fit *S* are based on *F*^2^, conventional *R*-factors *R* are based on *F*, with *F* set to zero for negative *F*^2^. The threshold expression of *F*^2^ > σ(*F*^2^) is used only for calculating *R*-factors(gt) *etc*. and is not relevant to the choice of reflections for refinement. *R*-factors based on *F*^2^ are statistically about twice as large as those based on *F*, and *R*- factors based on ALL data will be even larger.

### Fractional atomic coordinates and isotropic or equivalent isotropic displacement parameters (Å^2^)

**Table:**

	*x*	*y*	*z*	*U*_iso_*/*U*_eq_	
Br1	0.95523 (8)	0.14445 (5)	0.53910 (5)	0.03184 (18)	
Br2	0.50194 (8)	0.14394 (5)	0.54689 (5)	0.02883 (17)	
Br3	0.66511 (8)	0.39414 (4)	0.52626 (5)	0.02810 (17)	
Br4	0.73530 (8)	0.25471 (5)	0.29836 (5)	0.02880 (17)	
Cu1	0.71711 (9)	0.23115 (5)	0.48323 (6)	0.02398 (19)	
N3	0.3831 (6)	0.1546 (3)	0.1900 (4)	0.0200 (11)	
H3C	0.4761	0.1712	0.1585	0.024*	
N2	0.1266 (5)	0.0841 (3)	0.2321 (4)	0.0142 (10)	
C6	0.4153 (8)	0.0598 (4)	0.2422 (5)	0.0280 (14)	
H6A	0.4988	0.0658	0.3030	0.034*	
H6B	0.4505	0.0148	0.1912	0.034*	
C4	0.2717 (7)	0.1424 (5)	0.0866 (5)	0.0244 (14)	
H4A	0.3228	0.1055	0.0352	0.029*	
H4B	0.2425	0.2047	0.0551	0.029*	
C5	0.2642 (7)	0.0233 (5)	0.2805 (6)	0.0323 (16)	
H5A	0.2461	−0.0432	0.2587	0.039*	
H5B	0.2743	0.0264	0.3586	0.039*	
C1	−0.0752 (7)	−0.0431 (4)	0.2002 (5)	0.0236 (14)	
C2	−0.0285 (7)	0.0450 (4)	0.2597 (5)	0.0245 (14)	
H2A	−0.0182	0.0319	0.3366	0.029*	
H2B	−0.1112	0.0930	0.2431	0.029*	
N1	−0.1173 (7)	−0.1086 (4)	0.1523 (5)	0.0367 (14)	
C3	0.1243 (7)	0.0901 (5)	0.1130 (4)	0.0253 (14)	
H3A	0.0296	0.1241	0.0811	0.030*	
H3B	0.1212	0.0258	0.0826	0.030*	
C7	0.3101 (7)	0.2214 (4)	0.2631 (5)	0.0234 (13)	
H7A	0.3002	0.2852	0.2317	0.028*	
H7B	0.3771	0.2255	0.3323	0.028*	
C8	0.1493 (8)	0.1836 (4)	0.2783 (6)	0.0286 (15)	
H8A	0.0675	0.2259	0.2427	0.034*	
H8B	0.1390	0.1822	0.3546	0.034*	

### Atomic displacement parameters (Å^2^)

**Table:**

	*U*^11^	*U*^22^	*U*^33^	*U*^12^	*U*^13^	*U*^23^
Br1	0.0348 (4)	0.0360 (4)	0.0249 (3)	0.0130 (3)	0.0046 (3)	0.0072 (3)
Br2	0.0327 (4)	0.0263 (3)	0.0280 (4)	−0.0016 (3)	0.0064 (3)	0.0013 (3)
Br3	0.0331 (4)	0.0195 (3)	0.0339 (4)	0.0001 (3)	0.0121 (3)	0.0011 (3)
Br4	0.0297 (3)	0.0368 (4)	0.0197 (3)	0.0029 (3)	0.0026 (3)	0.0015 (3)
Cu1	0.0270 (4)	0.0230 (4)	0.0223 (4)	0.0029 (3)	0.0050 (3)	0.0014 (3)
N3	0.019 (2)	0.025 (3)	0.017 (3)	−0.003 (2)	0.006 (2)	−0.004 (2)
N2	0.017 (2)	0.012 (2)	0.013 (2)	0.0007 (19)	0.0032 (19)	−0.0025 (18)
C6	0.026 (3)	0.029 (3)	0.028 (4)	0.007 (3)	−0.001 (3)	0.003 (3)
C4	0.020 (3)	0.036 (4)	0.016 (3)	−0.009 (3)	0.002 (2)	0.000 (3)
C5	0.023 (3)	0.024 (3)	0.048 (4)	0.002 (3)	−0.003 (3)	0.012 (3)
C1	0.026 (3)	0.018 (3)	0.025 (3)	−0.005 (3)	−0.003 (3)	0.008 (3)
C2	0.025 (3)	0.024 (3)	0.026 (3)	−0.007 (3)	0.010 (3)	−0.002 (3)
N1	0.042 (4)	0.028 (3)	0.036 (3)	−0.012 (3)	−0.008 (3)	0.009 (3)
C3	0.023 (3)	0.045 (4)	0.008 (3)	−0.007 (3)	0.001 (2)	−0.002 (3)
C7	0.023 (3)	0.019 (3)	0.029 (3)	−0.004 (3)	0.005 (3)	−0.006 (3)
C8	0.040 (4)	0.015 (3)	0.035 (4)	−0.010 (3)	0.022 (3)	−0.013 (3)

### Geometric parameters (Å, °)

**Table:**

Br1—Cu1	2.3747 (11)	C4—H4A	0.9700
Br2—Cu1	2.4137 (11)	C4—H4B	0.9700
Br3—Cu1	2.3852 (10)	C5—H5A	0.9700
Br4—Cu1	2.3606 (11)	C5—H5B	0.9700
N3—C6	1.480 (8)	C1—N1	1.122 (8)
N3—C7	1.494 (7)	C1—C2	1.460 (8)
N3—C4	1.505 (7)	C2—H2A	0.9700
N3—H3C	0.9568	C2—H2B	0.9700
N2—C3	1.490 (7)	C3—H3A	0.9700
N2—C5	1.501 (8)	C3—H3B	0.9700
N2—C8	1.502 (7)	C7—C8	1.497 (8)
N2—C2	1.506 (7)	C7—H7A	0.9700
C6—C5	1.515 (9)	C7—H7B	0.9700
C6—H6A	0.9700	C8—H8A	0.9700
C6—H6B	0.9700	C8—H8B	0.9700
C4—C3	1.520 (8)		
			
Br4—Cu1—Br1	101.15 (4)	N2—C5—H5A	109.8
Br4—Cu1—Br3	97.32 (4)	C6—C5—H5A	109.8
Br1—Cu1—Br3	126.31 (4)	N2—C5—H5B	109.8
Br4—Cu1—Br2	123.02 (4)	C6—C5—H5B	109.8
Br1—Cu1—Br2	107.35 (4)	H5A—C5—H5B	108.3
Br3—Cu1—Br2	103.44 (4)	N1—C1—C2	176.7 (7)
C6—N3—C7	110.6 (5)	C1—C2—N2	111.8 (5)
C6—N3—C4	109.6 (5)	C1—C2—H2A	109.3
C7—N3—C4	109.4 (5)	N2—C2—H2A	109.3
C6—N3—H3C	106.5	C1—C2—H2B	109.3
C7—N3—H3C	122.3	N2—C2—H2B	109.3
C4—N3—H3C	97.4	H2A—C2—H2B	107.9
C3—N2—C5	109.8 (5)	N2—C3—C4	110.1 (5)
C3—N2—C8	108.5 (5)	N2—C3—H3A	109.6
C5—N2—C8	108.2 (5)	C4—C3—H3A	109.6
C3—N2—C2	110.8 (4)	N2—C3—H3B	109.6
C5—N2—C2	111.1 (4)	C4—C3—H3B	109.6
C8—N2—C2	108.4 (4)	H3A—C3—H3B	108.2
N3—C6—C5	108.9 (5)	N3—C7—C8	108.6 (5)
N3—C6—H6A	109.9	N3—C7—H7A	110.0
C5—C6—H6A	109.9	C8—C7—H7A	110.0
N3—C6—H6B	109.9	N3—C7—H7B	110.0
C5—C6—H6B	109.9	C8—C7—H7B	110.0
H6A—C6—H6B	108.3	H7A—C7—H7B	108.4
N3—C4—C3	107.8 (5)	C7—C8—N2	110.3 (5)
N3—C4—H4A	110.1	C7—C8—H8A	109.6
C3—C4—H4A	110.1	N2—C8—H8A	109.6
N3—C4—H4B	110.1	C7—C8—H8B	109.6
C3—C4—H4B	110.1	N2—C8—H8B	109.6
H4A—C4—H4B	108.5	H8A—C8—H8B	108.1
N2—C5—C6	109.2 (5)		

### Hydrogen-bond geometry (Å, °)

**Table:**

*D*—H···*A*	*D*—H	H···*A*	*D*···*A*	*D*—H···*A*
N3—H3C···Br3^i^	0.96	2.62	3.420 (5)	142
N3—H3C···Br2^i^	0.96	2.95	3.545 (5)	122
C4—H4A···Br3^i^	0.97	2.92	3.555 (6)	124
N3—H3C···Br4	0.96	2.86	3.406 (5)	117
C2—H2A···Br1^ii^	0.97	2.91	3.638 (6)	132
C2—H2B···Br4^iii^	0.97	2.73	3.608 (6)	150

Symmetry codes: (i) *x*, −*y*+1/2, *z*−1/2; (ii) −*x*+1, −*y*, −*z*+1; (iii) *x*−1, *y*, *z*.
